# Formative evaluation and adaptation of pre-and early implementation of diabetes shared medical appointments to maximize sustainability and adoption

**DOI:** 10.1186/s12875-018-0797-3

**Published:** 2018-07-07

**Authors:** Christine P. Kowalski, Miranda Veeser, Michele Heisler

**Affiliations:** 1 Center for Clinical Management Research (CCMR- VA), VA Ann Arbor Healthcare System, 2800 Plymouth Road Bld. 16, Ann Arbor, MI 48109 USA; 2 Department of Internal Medicine, University of Michigan, 1500 East Medical Center Drive, Ann Arbor, MI 48109 USA

**Keywords:** Formative evaluation, Implementation, Diabetes, Shared medical appointments, Qualitative research, Facilitation, CFIR, Adaptation

## Abstract

**Background:**

Understanding the many factors that influence implementation of new programs, in addition to their success or failure, is extraordinarily complex. This qualitative study examines the implementation and adaptation process of two linked clinical programs within Primary Care, diabetes shared medical appointments (SMAs) and a reciprocal Peer-to-Peer (P2P) support program for patients with poorly controlled diabetes, through the lens of the Consolidated Framework for Implementation Research (CFIR). We illustrate the role and importance of pre-implementation interviews for guiding ongoing adaptations to improve implementation of a clinical program, achieve optimal change, and avoid type III errors.

**Methods:**

We conducted 28 semi-structured phone interviews between September of 2013 and May of 2016, four to seven interviewees at each site. The interviewees were physician champions, chiefs of primary care, pharmacists, dieticians, nurses, health psychologists, peer facilitators, and research coordinators. Modifiable barriers and facilitators to implementation were identified and adaptations documented. Data analysis started with immersion in the data to obtain a sense of the whole and then by cataloging principal themes per CFIR constructs. An iterative consensus-building process was used to code. CFIR constructs were then ranked and compared by the researchers.

**Results:**

We identified a subset of CFIR constructs that are most likely to play a role in the effectiveness of the diabetes SMAs and P2P program based on our work with the participating sites to date. Through the identification of barriers and facilitators, a subset of CFIR constructs arose, including evidence strength and quality, relative advantage, adaptability, complexity, patient needs and resources, compatibility, leadership engagement, available resources, knowledge and beliefs, and champions.

**Conclusions:**

We described our method for identification of contextual factors that influenced implementation of complex diabetes clinical programs - SMAs and P2P. The qualitative phone interviews aided implementation through the identification of modifiable barriers or conversely, actionable findings. Implementation projects, and certainly clinical programs, do not have unlimited resources and these interviews allowed us to determine which facets to target and act on for each site. As the study progresses, these findings will be compared and correlated to outcome measures. This comprehensive adaptation data collection will also facilitate and enhance understanding of the future success or lack of success of implementation and inform potential for translation and public health impact. The approach of using the CFIR to guide us to actionable findings and help us better understand barriers and facilitators has broad applicability and can be used by other projects to guide, adapt, and improve implementation of research into practice.

**Trial registration:**

ClinicalTrials.gov ID: NCT02132676.

## Background

Understanding the many factors that influence implementation of new programs, in addition to their success or failure, is extraordinarily complex. Formative evaluation, defined as a rigorous assessment process designed to identify potential and actual influences on the progress and effectiveness of implementation efforts, is an essential means to systematically approach this complexity [1].

Many implementation studies rely exclusively on summative data-- or data outputs, products, and outcomes—to determine program success or failure. While summative data is useful, it is not adequate to understand critically important implementation processes [1, 2]. When used in isolation, summative data often leads to the term “implementation black box” [3]: there is no way to understand the specific reasons the intervention succeeded or failed, how it was actually implemented, and how local contextual factors affected implementation. This is where formative evaluation comes in, to fill in these gaps and to systematically examine key features of the local implementation setting, detect and monitor unanticipated events and adjust if necessary in real-time, optimize implementation to improve potential for success, and avoid type III errors—the failure to detect differences between the original intervention plan and the ultimate manner of implementation that lead to failure to achieve outcomes [1, 4]. This understanding is essential for efforts to sustain, scale up, and disseminate any new program–otherwise there is potential for failure to account for specific contextual issues in program implementation.

Adaptations have been found to be necessary for sustainable implementation [5] and are considered part of the traditional translation pipeline (adaptations as required) [6]. Progress has been made in advancing the science of implementation, but too often the complexity of translating research into practice is overlooked or follows an overly simplistic model [7]. In the words of implementation experts Dr. Chambers and Dr. Norton, “Rather than assuming that adaptation of a manualized intervention is at odds with good implementation, the field can systematically collect information on the impact of adaptation to individuals, organizations, and communities and use this information to extend the knowledge base of implementation of evidence-based practices as well as ongoing improvement of the evidence-based practices themselves” [8]. Indeed other efforts in implementation have been described where adaptations are catalogued on an ongoing basis. [9] While the static view of adaptation has been that it is bad or to be avoided and/or eliminated, the dynamic sustainability model believes adaptation to be “inevitable and encouraged.” [7] Likewise, simplified intervention implementation overlooks the complexities of translating research to practice and relies on a set of assumptions that limits enhancement of fit between evidence-based interventions and delivery setting. [10]

Additionally, while the evidence-to-practice gap for interventions is receiving attention, it tends to be understudied in primary care. [11] A recent study highlighted the importance of paying attention to context, which was noted as frequently failing to be acknowledged, described, or taken into account during implementation in primary care. [11]

Accordingly, to fill this gap in the literature, we sought to illustrate the role and importance of pre-implementation (early) interviews for guiding ongoing adaptations to improve implementation of a clinical program, achieve optimal change, and avoid type III errors. We gathered detailed pre-implementation data across five health system sites, within primary care settings, that had each committed to institute Shared Medical Appointments (SMAs) and in some SMA cohorts an additional offered mutual peer support program (P2P) for adult patients with poorly controlled diabetes. In particular, we examined modifiable barriers and facilitators to implementation. We then documented the adaptations that were made in real-time to attempt to improve implementation. This comprehensive data will also facilitate and enhance understanding of the future success or lack of success of implementation of new innovative clinical programs such as SMAs and inform potential for translation and public health impact of these [2].

## Methods

This qualitative study is part of a larger implementation study, “The Shared Health Appointments and Reciprocal Enhanced Support (SHARES) study” [12]. The SHARES study is a multi-site cluster randomized trial of five geographically diverse Veterans Affairs (VA) health systems evaluating the effectiveness and implementation of diabetes Shared Medical Appointments (SMAs) with and without an additional reciprocal Peer-to-Peer (P2P) support program, when compared to usual care.

SMAs bring patients with the same chronic condition together with an interdisciplinary team of providers to provide shared education and support. The diabetes SMAs consist of a series of 1–2 h sessions of 8–10 patients led by a team of health professionals. At each site, participants have a total of approximately 8 h of sessions, and the sessions are intended to be interactive and focus on key diabetes self-management topics (e.g., diet, medications, physical activity, self-monitoring). The P2P program comprises periodic peer support, [13, 14] group sessions, and telephone contact between SMA participant pairs to promote more effective diabetes self-management and sustain gains achieved through the SMAs after completion of these sessions. Outcomes will be examined across three different treatment groups: (1) SMAs; (2) SMAs plus P2P; and 3) usual care.

We undertook a type of formative evaluation (FE), labeled implementation-focused evaluation, of the SMAs and of P2P [1]. This type of FE occurs throughout implementation of the project plan: before, during, and after implementation. This manuscript focuses on the real-time FE that took place during the early stages of implementation and pre-implementation.

The overarching framework for the SHARES qualitative discovery-- including the interviews, formative evaluation, implementation, and analysis-- was the Consolidated Framework for Implementation Research (CFIR) [15]. The CFIR provides a framework of 39 constructs from across published implementation frameworks that describe the organizational and contextual setting and are believed to influence implementation. We are tracking the CFIR constructs across the sites throughout the project and will ascertain how they influence implementation success during future program evaluation.

Institutional Review Board (IRB) approval was obtained from the Central IRB.

### Data collection and analysis

A brief phone survey was conducted with key informants from each site to identify potential key CFIR constructs. We then conducted 28 semi-structured phone interviews with participants at five VA health systems, four to seven interviewees at each site (see Table 1). Interviews were conducted between September of 2013 and May of 2016 and lasted between 22 and 56 min (mean of 34 min). Two interviewers participated in all interviews; detailed notes were taken by both, while most of the questions were asked by the first interviewer. The interviewees were physician champions, chiefs of primary care, pharmacists, dieticians, nurses, health psychologists, P2P facilitators, and research coordinators from the local sites. After each initial interview, the interviewee was then asked to recommend other informants. This type of recruitment, called snowball sampling, means that rather than determining individuals to interview ahead of time, we asked everyone that we interviewed to recommend other potential participants, including key clinical opinion leaders. Interviewees from each site were continually recruited until the research team felt that qualitative data discovery had reached saturation—the point at which new data only confirmed the themes and conclusions already achieved [16].

**Table 1:** Qualitative interviewee titles

Job title of interviewee	Site 1001	Site 1002	Site 1003	Site 1004	Site 1005	*n* = 28
	*n* = 6	*n* = 6	*n* = 5	*n* = 7	*n* = 4	
Clinical pharmacist (PharmD)	1	1	1	3		6
Primary Care physician	1	1	1		2	5
Nurse Manager	1	1				2
Health Psychologist (PhD)	2		1			3
Research Coordinator	1			1		2
P2P group facilitator			1	1		2
Chief of Cardiology				1		1
Chief of Primary Care				1		1
Dietician			1		1	2
Associate Chief of Staff					1	1
Chief of Ambulatory Care		1				1
Nurse		2				2

Phone interviews were qualitative and, unlike a survey, all questions were open-ended; interviewees were encouraged to share their experiences in detail to enable a thorough understanding of the implementation experience from their varying perspectives. Conducting multiple interviews at each site enabled us to understand how perspectives compared from staff in different positions.

Our interview guide included questions about the interviewee’s role in the diabetes SMAs and P2P groups, aspects of the program they would like to change, barriers and facilitators with regards to implementing/expanding the diabetes SMAs and P2P programs, what kinds of resources or tools they would need for implementation, need for and awareness of evidence for the P2P program in addition to SMAs, existence of a clinical champion or local opinion leader, and types of feedback they would like to receive as the study progressed. Although CFIR-relevant questions were asked, there were opportunities to explore non-CFIR issues in that the interviewees could discuss anything related to their implementation experience, which was described by the participants in detail. After each interview, we developed a list of barriers, facilitators, and tasks that we needed to accomplish or follow up on with key staff at each of the local sites as part of the implementation process.

The initial lists of barriers and facilitators were developed from the detailed interview notes with a quick turnaround time to implement an immediate feedback loop to the sites because the primary consideration was not collecting data for “research,” but rather to implement adaptations to overcome barriers and improve the implementation process. After these barriers were reviewed, the implementation team worked with the local staff from each site to make adaptations as necessary. Of note, some of the adaptations were driven by local clinical staff (see Table 2 for these details). We shared feedback with each local site continually while the phone interviews progressed so that suggestions could be incorporated in a timely manner. Each of these items was addressed to the extent that it could be prior to a mid-implementation site visit. For any barriers that we had not been able to address before each site visit, a detailed summary packet was written by the project qualitative analyst (CPK) and distributed to the implementation team members attending the site visit, with background information and a list of questions and tasks that still needed to be addressed. In addition, conference calls with individual sites occurred throughout this study period and detailed notes about important issues were documented and included in analysis.

**Table 2:** Detailed adaptations made in real-time by site and CFIR construct

Construct	Barrier	Facilitator or Adaptation
INTERVENTION CHARACTERISTICS		
Intervention Source Definition: Perception of key stakeholders about whether the innovation is externally or internally developed.		1001: Internal
		1002: External
		1003: Internal
		1004: External
		1005: Internal
Evidence Strength & Quality Definition: Stakeholders’ perceptions of the quality and validity of evidence supporting the belief that the innovation will have desired outcomes.		1001:
		• Interviewees see benefit in social support that SMA and P2P programs would provide. This was also shown in their trial program.
		• See also, Trialability.
		• See also, Knowledge and Beliefs.
	1002:	1002:
	• Repeated throughout all the interviews, that the SMAs and P2P programs are seen as extra work and staff do not see any added value – overall negative mindset to this implementation.	• Our team presented evidence during the site visit and during a local primary care team meeting to help educate and influence the physicians.
	• Diabetes SMAs have been a hard sell with physicians; they are not on board – they do not see an added value.	1003:
		• “Buddy system” (similar to P2P) has been effective in other settings in the facility, which has in turn increased support for P2P program.
		• HBC believes evidence behind SMAs is good and they were already looking for opportunities to improve their diabetic population outcomes. Believes the SMA and P2P will be very “*fruitful and helpful*.”
		• Physicians involved believe they have seen evidence that having a peer or buddy for support will help the diabetic population.
		1004:
		• Leadership is on board and thinks there is good evidence for the positive effect of being part of a group for the SMAs and the P2P components.
		• See also, Knowledge and Beliefs.
		• See also, Leadership Support.
		1005:
		• The ACOS for Ambulatory Care believes strongly in the evidence for SMAs.
		• SMA PCP feels there is evidence that the SMA and P2P will engage the patients (participants); feels they are more motivated by hearing from peers than from a clinician- belief of local evidence that the group portion of the SMA and the P2P group will be beneficial.
		• See also, Knowledge and Beliefs.
		• See also, Leadership Support.
Relative advantage Definition: Stakeholders’ perception of the advantage of implementing the innovation versus an alternative solution.	1001:	1001:
	• According to some interviewees, staff were not encouraging their patients to attend the SMAs because they did not see an advantage of the SMAs compared to usual care.	• We had local staff present information about the SMA program and the value of it to the PCPs.
		• Our team presented evidence during the site visit and during a local primary care team meeting to help educate and influence the physicians.
	1002:	
		• “Don’t see how it could hurt” attitude.
	• Physicians see the diabetes SMAs as extra work; do not see the added value. • See also, Evidence Strength & Quality.	1002: • Our team presented evidence during the site visit and during a local primary care team meeting to help educate and influence the physicians.
		• Nursing staff seems to be on board – believe in peer support aspect to improve diabetes care/outcomes for Vets. Believe Veterans listen to peers more than clinicians.
		1003:
		• Have had other diabetes studies at site, but group support was not formalized, “no mechanism for patients who have been-there-done-that providing support to others.”
		• Health psychologist and other staff saw SMAs as an advantage to their already mandated diabetes education classes because they had not translated into any action.
		1004: • Staff see a need for peer mentoring program in Veterans especially because they are deployed in a unit and relate to their Veteran peers.
		• A physician leader thinks that there may be a financial benefit to the SMA group and P2P component.
		• See also, Cost. 1005: • The Associate Chief of Staff sees the advantage of the diabetes SMAs because he thinks it will help with access, efficiency, and help Veterans to learn from each other. • Currently, the ACOS says there are 5–6 separate patients meeting with the clinical pharmacist specialist for 30 min each going over the same information with some tweaking for their condition. • Some PCPs talked about how they could see the relative advantage of doing group visits vs. one on one patient visits. • See also, Cost.
	1005: • Possible added work for clinicians due to number of patients needing clinical notes following SMAs; described as: *“It is a little bit of extra work because I have to write you know, 8 to 12 notes rather than just the four that I would write in two hours, but it potentially helps, but, as a doc, that[‘s] the biggest detriment I see to it.”* • While PCP SMA lead is excited about the prospect of group visits, there was only a 50/50 excitement from other PCPS at this site for expansions of SMAs. Levels of enthusiasm varied because some PCPs simply like the idea of group appointments and some do not.	
Adaptability	1001:	1001:
Definition: The degree to which an innovation can be adapted, tailored, refined, or reinvented to meet local needs.	• Concern from staff if the program was not adaptable and patients may not want to work with their assigned partner and this may cause them to leave the study.	• We worked with the site to come up with an adapted plan whereby a patient who does not work well with his/her partner can be re-paired or put into a group of 3. We also worked with the nurses and PCPs for their recommendation on patients that will work well together. The site appreciated us working with them to make the peer pairing adaptable.
	• Several staff were concerned that the locally designed recruitment plan was too ambitious.	
	• Nurses originally going to take charge of SMAs, varying levels of comfort and would need to train too many facilitators.	
		• During a pre-implementation local site visit our project staff discussed recruitment; the site did change their recruitment strategy to be more realistic.
	1002:	
	• Staff here were concerned with the standardization that they perceived was required of their local SMAs. This site did not feel the program was very adaptable initially.	• HBC or psychology fellow to fill role of nurses as leaders of SMAs.
		1002: • We were able to work with the local team through meetings and calls to ensure that the SMAs could be adapted as each site saw fit. Each sub-site was able to come up with its own SMA plan.
		1004: • Site has tailored current SMA visits according to Veteran feedback so they can get what they want out of sessions.
Trialability Definition: The ability to test the innovation on a small scale in the organization, and to be able to reverse course (undo implementation) if warranted.		1001: • Pilot SMA was conducted before our clinical program began. Local staff involved decided it was too difficult for patients to absorb all the information in a one-day SMA. Also, because they were making meta-adjustments/medication changes, they felt the sessions needed to be longitudinal to titrate. *“We feel we can’t fix all of that in just one visit.”* 1002: • Piloted SMAs locally prior to implementation. 1005: • Piloted SMAs locally prior to implementation.
Complexity = 4 Definition: Perceived difficulty of the innovation, reflected by duration, scope, radicalness, disruptiveness, centrality, and intricacy and number of steps required to implement.	1002: • Staff are “busy and stretched thin;” it is difficult to do anything additional. • Contrary to all our other sites we were told that the amount of training for the peer facilitator needed to be minimal. They had trouble finding a P2P facilitator because of their perception of the work required. • Originally staff felt this project was only supposed to be adding on the P2P component. “Yet, somehow it has ended up to be a lot more work for the SMA people.” Staff feel they had to make multiple changes to the SMAs that they were not anticipating. • Staff expressed annoyance about the work involved and administrative tasks: “very frustrating,” “just more stress.” The timeline getting pushed back “just became unnerving.” They did not anticipate that this project “would be so much work.” 1003: • Staff told us their largest barrier is always funding and finding time in staff schedules to devote to this project. 1004: • Very busy staff and many competing initiatives; not only diabetes, but overall information overload. 1005: • Although the ACOS is very supportive of the SMAs he did say that it cannot add extra work to his employees. • Clinical pharmacist notes are a large barrier. Generally, clinical pharmacist notes are very comprehensive and they are the SMA documenters for this site. ACOS is concerned about the amount of time the documentation of the diabetes SMAs will take for the clinical pharmacists. He wants someone to make sure that we build thoughtful templates that capture what is taking place, but for the most part are standard curriculum.	1001: • Interestingly, this is the only site where we did not hear about staff being overly busy, stretched thin. 1002, 1003, 1004, 1005: • Facilitation team worked with the sites through team meetings and phone meetings to streamline documentation, shared diabetes SMA clinical note templates across the sites, worked to better integrate into existing workflow with input from staff.
Design Quality and Packaging Definition: Perceived excellence in how the innovation is bundled, presented, and assembled.	All sites: We added this because of the comments from all sites that clinical staff was not always aware of what was happening and what the intervention actually was –many of the staff did not understand what P2P was and we spent a good portion of the interviews explaining P2P. This was not really the intention of the interviews going in, but we spent a lot of time on clarifications and answering questions.	
Cost Definition: Costs of the innovation and costs associated with implementing the innovation including investment, supply, and opportunity costs.	1002: • Nursing leadership has suggested cost, in terms of staff time for the P2P facilitators, as a barrier to implementation of this program.	1004: • A physician leader thinks that there may be a financial benefit to the SMA group and P2P component.
		1005: • The ACOS feel that SMAs should improve efficiency of care and access.
Outer setting		
Patient Needs & Resources Definition: The extent to which the needs of those served by the organization (e.g., patients), as well as barriers and facilitators to meet those needs, are accurately known and prioritized by the organization.	1001: • Patients have pre-paid phones, run out of minutes and are not able to make calls at the end of the month. • Patients are “guarded” and may not want to share phone numbers with their peer. • Clinicians stated top barrier would be “convincing the patients to show up.” • Patients not motivated in general to come to appointments or sessions unless compensated financially. • Lack of patient motivation or follow-though. • Low patient attendance to SMAs and P2P drop-ins. • Concern that copay could contribute to poor attendance. • Concern about early morning start – some Veterans come in without eating before, which leads to very low blood sugars. • Delays to the start of the diabetes SMAs; knowing when the patients arrive and where to take vitals (time). • Plan for post-SMA continuation of care. 1002: • Difficulties with patient recruitment for SMAs; believe they will experience the same problem for the P2P groups. Lack of motivation among patients to attend. • Local patients are elderly and very private. May not want to work with a peer; concern about potential mis-matches alienating patients from participating. • Concern about distance – many Veterans live far from this VA. • Concern Veterans may not want to stay after the SMA for P2P. 1003: • Patient attendance/compliance is low—particularly among patients with A1cs over 9. Elderly population with less financial means; many do not have phones or access to the internet. • Patients resistant to change. • Concerns from multiple staff about “passive patient population” through experience with SMAs patients tend to be passive and expect you to do something rather than making a change for themselves. • Some patients prefer not coming to clinic unless they will receive travel pay. • Concerns about attendance due to ongoing construction**.** 1004: • All interviewees mentioned that it is difficult to get buy-in from patients to participate in groups. • In their experience with recruiting for the diabetes SMAs you need to recruit 3 patients for every 1 who attends. • VA does not reimburse patients for travel to research visits. Sometimes patients would like to join the groups but cannot afford to travel without compensation. • Many patients at this site use disposable phones so their phone numbers frequently change. Nurses have this experience when they try to call patients for reminders. • Some Veterans work and take classes, making timing/attendance difficult.	1001: • The local RA facilitated patient attendance through reminder calls and letters. • We worked with the local nurses to determine how to ensure staff are better aware of when the patients arrive for SMAs to ensure a timely start. • Moved SMA start time to early in morning to resolve parking issue and in hopes to increase attendance. • Studied barriers to attendance – poor attendance correlated with adherence issues. • Adapted so that the SMA is no longer one full day. This was done to allow time for medication adjustments, which could not be done when the SMA as only 1 day. • We consulted with staff from the site to take into account their perspective on matching peers together and who would work best together allowing for adaptability and patient re-pairing. • We also worked with site to ensure whenever possible that these facilitators would be sustainable across time when the research team would no longer be involved (transference of some of these tasks in time to local clinical and administrative staff). • Worked to guarantee eligibility for travel pay for SMAs. 1002: • The local RA facilitated patient attendance through reminder calls and letters. • Presentations were given at staff meetings to increase patient attendance/ referrals. • We worked with staff from the site to consider their perspective on matching peers together and which would work best together (taking into account disease state, gender, age). • Worked to guarantee eligibility for travel pay for SMAs. • As above worked with site to ensure sustainability of facilitators. 1003: • The local RA facilitated patient attendance through reminder calls and letters. • Word of mouth support from Veterans who have participated in the diabetes SMAs to other Veterans has helped. This has been mostly serendipitous rather than organized. We discussed this with the local site PI and she presented information on P2P to a Veteran-run wellness group to help with the word-of-mouth support. • We instituted a way to distribute reminders for the P2P groups. • We worked to make sure the initial group script is very dynamic. • Worked to guarantee eligibility for travel pay for SMAs. • SMAs have been modified to better fit patient needs (number of sessions, etc.) • Veterans appreciate having an interdisciplinary team to guide them. • Social support will increase patient accountability/attendance. • As above worked with site to ensure sustainability of facilitators. 1004: • The local RA facilitated patient attendance through reminder calls and letters. • One facilitator staff has noticed is having 2 health psychologists participate in the SMAs to make sure that patients’ needs and wants are addressed in the class and moving the sessions to more of a conversation rather than a didactic session—has already been successful. • Vets will benefit from added social support and “hearing from ‘equals’ rather than somebody else.” • Worked to guarantee eligibility for travel pay for SMAs. • As above worked with site to ensure sustainability of facilitators. 1005: • The importance of goal setting, as a patient need, was discussed in regards to prior SMAs and how that was needed to improve outcomes – being held accountable helps to improve patient outcomes. • The local RA facilitated patient attendance through reminder calls and letters. • Social support is seen as a patient need and the SMA and P2P groups will fill a gap in patient needs. • PCPs here view the well-controlled patients attending the SMAs as a facilitator. • Worked to guarantee eligibility for travel pay for SMAs.
INNER SETTING		
Structural Characteristics Definition: The social architecture, age, maturity, and size of an organization.		1005: • There is a new patient education room with the exam room attached. This site was getting ready to ramp up the SMA model. “*We are well situated to make this work*.”
Networks /Communications Definition: The nature and quality of webs of social networks, and the nature and quality of formal and informal communications within an organization.	1002: • Concern that there would be a communication gap between the SMA coordinator and the P2P facilitators. • Very large project and having 3 local sites makes it even more complicated because each site has some differing challenges. • Communication between main study site and 1002 cited as problematic. • Staff can be difficult to reach and get in contact with. • Concern word still needs to be spread about project. • The overall project site staff has ongoing difficulties in communicating with this site.	1001: • Nurse that managed previous SMAs has well established relationships with key stakeholders. • PharmD & health psychology fellow offered and gave more information to physicians at staff meetings. • Keeping project on MDs minds will help with referrals to program, so staff ensure this was done. • Champion is also chief – runs primary care meetings and encourages support from physicians. 1002: • We worked with this site and held team conference calls and developed a plan to address communication. • A staff member who knows the patient panels well is working to communicate with and enlist her providers.
Culture Definition: Norms, values, and basic assumptions of a given organization.	1001: • Multiple staff describe this site as a culture of Veterans not wanting to participate in group settings. • Veterans here are very “guarded” and have culture of not being very motivated to make their own changes, do not bring back homework, do not bring in things asked to bring. 1002: • Culturally have great difficulty getting staff to commit a few hours a week to any type of project, even though this site has more financial resources than others. The site is still very cautious to commit staff; they will not commit to having a pharmacist attend the SMAs unlike all other sites. There is also a reluctance to write down responsibilities because of a fear they will become an expectation.	
Tension for change Definition: The degree to which stakeholders perceive the current situation as intolerable or needing change.	1003: • Diabetes education classes have been mandated, but have not “translated into action.”	1001: • This site has a lack of group appointments, interviewees see need for program that will provide extra social support. 1003: • SMAs seen as potential solution to lack of action/improvement in diabetes management. • Staff see need for innovation at their facility. 1004: • “Benefit to hearing from ‘equals’ rather than somebody else – someone lateral as opposed to top down…” • Always looking for new programs to help their “frequent flyers.”
Compatibility Definition: The degree of tangible fit between meaning and values attached to the innovation by involved individuals, how those align with individuals’ own norms, values, and perceived risks and needs, and how the innovation fits with existing workflows and systems.	1002: • Had mixed drop-in sessions which they now cannot do with diabetes SMAs. 1004: • According to the chief of primary care, it is very important that this process fit into the existing workflow for implementation to succeed. • It is very important that we foster buy-in from frontline providers and they need to see this [P2P] as integrated and not imposed or there will be pushback. We need to coordinate our work into the suite of already existing programs.	1001: • Local staff have confidence that research implementation will be smooth at facility because it will fit within existing programs. • This innovation is considered by staff to be good compliment to what is already going on in patient care. • Not a lot of diabetes programming, fits need. 1002: • SMAs were already in place at this site and P2P perceived as easy to add on. 1003: • Intervention fits within existing structure, some minor changes able to be made with information provided by innovation staff. 1004: • Chief of primary care presented information about P2P at a monthly staff meeting. • We spoke with several front-line staff about how to best integrate this with their already existing work. • The Director of primary care sent emails about the project. Coming from him will help elevate the status of the project. • The Director of primary care will present information at the bi-weekly meetings with team leaders. • Diabetes groups already running, innovation will be able to fit within context of ongoing groups.
Relative Priority Definition: Individuals’ shared perception of the importance of the implementation within the organization.	1002: • Intervention not on leadership radar – voluntary, so can be first of things to go. 1004: • The top barrier was stated to be infringement on their previous initiatives. During the interviews, we determined that if the staff already involved in diabetes management feel their work is being challenged or re-directed by P2P it will “put their backs up.” • Very busy staff and many competing initiatives; not only diabetes, but overall information overload.They	1001: • Leadership thinks the SMA expansion will easily fit in because already had SMAs ongoing. 1002: • Nurses want program to be success – trying to enlist more people/rally support. 1004: • The qualitative interviews with front line nurses and physicians helped determine how to integrate P2P with their existing workflow and programs. We also asked for their suggestions for modifications to enable local success and gain buy-in. • Chief of primary care circulated info to those involved in a strategic planning initiative to let them know what will be happening and how to incorporate it. • To overcome sense of infringement the chief of primary care suggested 3 people as potential champions and said it was very important for us to get them on board: 1) a diabetes management nurse, who is the “epicenter of things” and the “clearing point” for diabetes management, 2) the acting chief of pharmacy, who oversees the clinical pharmacists in primary care, and3) a highly-engaged dietician. We interviewed all three to get their perspective and pull them into the study. • We sent the Chief of primary care a summary to circulate to those involved in the strategic planning initiative so all can be on same page, let them know what’s coming and how to incorporate it. He pulled together a distribution list.
Readiness for implementation Definition: Tangible and immediate indicators of organizational commitment to its decision to implement an innovation.		1001: • This site is ready for implementation: the plan for when study related SMAs will begin is in place – recruitment strategies, SMAs already running and have been through trial and error period.
Leadership Engagement Definition: Commitment, involvement, and accountability of leaders and managers with the implementation of the innovation.	1002: • Leadership engagement is lacking compared to the other 4 sites. Physicians are not engaged or supportive of SMAs and the P2P groups. The leader who is the chief for one site and our local PI was mentioned as not being influential by several local staff. This could be complicated by the fact that there are 3 local sites. • Also, there was a barrier discussed confidentially by multiple staff about a high level leader being a barrier for this project, specifically, as well as other projects. This person is not supportive and has blocked nurses from being involved in this as well as other projects. Solving this issue was beyond the scope of our project.	1001: • We were impressed with Chief of primary care as are local staff. Helped to convince providers to enroll patients, blocked out time for clinicians to be at groups, guaranteed space, and made sure the project ran smoothly overall. • At this site we have support from leaders across disciplines. • General support for SMAs from staff. 1002: • Multiple interviewees said staff and clinicians are aware these programs. 1003: • Interviewees suggest leadership engagement is present and they have leadership support for the SMAs and P2P. This came from multiple staff including physicians. 1004: • Very impressive chief of primary care. Was very thoughtful in his remarks and what will need to be done on his behalf for this program to succeed. Several other staff also mentioned his as a very supportive leader who is engaged in this study. • Chief of primary care offered to help us to make sure staff see P2P as a benefit, leading from a high level, tell others why we are implementing P2P and that this is important work. • Director of primary care said data feedback is important for his staff to stand behind this and he offered to disseminate data to the strategic initiative quad and everyone involved in diabetes care. 1005: • We were very impressed with the ACOS for Ambulatory Care. He is extremely supportive of the diabetes SMAs and pushing them forward and making sure PharmDs are able to participate despite time constraints. He sees an advantage to having the SMAs in terms of efficiency. • See also, Relative Advantage and Knowledge and Beliefs.
Available resources Definition: The level of resources organizational dedicated for implementation and on-going operations including physical space and time.	1001: • Space constraints for group visits. • Rooms have been scheduled for SMAs, but when patients arrive that space is occupied. • Patient parking is often not available. • Psychologist who was the P2P facilitator and SMA facilitator was not renewed and now they must find someone new and re-train. • Not all resources are available for getting patients checked in and vitals taken prior to SMAs. For example, need their own scale. 1002: • Space is so limited that groups here are scheduled based on room availability rather than staff availability. • Facility covers a large geographic area; some Veterans live 200 miles away from their facility. • Cost concerns have meant that they use volunteers for the P2P facilitator position. This caused concern that they may not be here at the right time or not have the right skills to serve as the P2P facilitator. Indeed there was P2P facilitator turnover and re-training required. • The amount of training for the peer facilitator really needs to be minimal. We did not hear this from the other sites. • Staff are “busy and stretched thin;” it is difficult to do anything additional. 1003: • Patient parking is lacking and there are construction projects ongoing. • Group meeting space is constrained (SMAs and P2P drop-in). • Staff told us their largest barrier is always funding and finding time in staff schedules to devote to this project. 1004: • Time demands on staff are a major issue. We were told that if a lot of time would be required for patient recruitment and screening that implementation would be very difficult. • Busy staff, competing initiatives; overall information overload. • Space constraints for group meetings. • Concern there will not be enough resources to continue program after study period ends (SUSTAINABILITY). • Parking can be issue. 1005: • There is a big issue with lack of resources at this site. This was seen in terms of clinical pharmacist leaving and they were not able to replace her and the resulting lack of time for the remaining pharmDs. This may be related to the insistence that the SMAs be kept team specific. This issue came up at local team meetings with pharmacists and dieticians. • From the ACOS, *“We are short-staffed right now and we are unable to hire people.”*	All sites: • Worked to find a guaranteed room (applies to all except 1005). • Scheduled out all rooms for SMAs and P2P open group sessions in advance. • P2P phone access to peers is available and always a viable option for all. • We did write scripts for the P2P facilitators to ease their workload and make it easier to understand their role. We also hosted bi- monthly training and question and answer sessions for them to talk to the facilitators as well as all other site P2P facilitators. 1003: • Made sure the classroom was reserved early for next couple of years for P2P. • Likely patient parking/construction problems will be resolved by time funding comes through. 1004: • We worked with the local staff to ensure that the P2P process will fit into the existing workflow. Additionally, P2P was presented at a monthly staff meeting by study staff. • Director of primary care offered to help us to make sure staff see this as a benefit, leading from a high level, tell others why we’re doing P2P, ‘this is important work.’ • We will send ongoing data to the director of primary care and he will disseminate to the strategic initiative quad and everyone involved in diabetes care. • RA will help to relieve time demands of staff for implementing initiative 1005: • The ACOS is very on board (see also Leadership) and did help to overcome some of these barriers, such as securing time from the PharmDs despite their initial statements that they did not have enough time. However, see his caveat at left.
Access to knowledge &information Definition: Ease of access to digestible information and knowledge about the innovation and how to incorporate it into work tasks.	1001: • Chief of Primary Care aware of study. 1002: • Extended project delays (over a year) and the roll-out keeps getting pushed back with a lot of time to not know what is happening has made staff uncomfortable. Other key stakeholders may not be aware of project because of these delays – leadership has not pushed it due to delays. • Lack of awareness of project	1001: • PharmDs speak at primary care meetings to educate MDs about SMA groups. • In general, most staff aware of the way study will be conducted. 1004: • P2P facilitator engaged and knowledgeable of her role.
CHARACTERISTICS OF INDIVIDUALS		
Knowledge & Beliefs about P2P and SMA Definition: Individuals’ attitudes toward and value placed on the innovation, as well as familiarity with facts, truths, and principles related to the innovation.	1001: • Staff mentioned that they are curious to see if this P2P has an added benefit to the SMAs. Kind of a wait and see how it goes approach more than already believing in the evidence. • Unclear how much clinicians know about P2P aspect of program. • Some confusion over how patients will be communicating. • Some concern about patients sharing incorrect medical information. 1002: • Some staff were worried about the P2P group and its purpose. They thought the patients would be giving incorrect clinical advice to each other. • Some staff generally confused about the way implementation/P2P would work. 1004: • Some staff questioned aspects of the P2P study evaluation such as the patients recalling and self-reporting the number of times they had spoken with their peer partner (for those not using the telephone system). They do not think patients will be able to accurately recall. 1005: • Concern was common at this site that the patient may give each other incorrect clinical information when paired up in peer-to-peer. • The dietician discussed a negative belief about the interaction of peers and how one could be over-bearing and change the tone. • PCP interviewee talked about his beliefs that it may be possible that the pairing might not be well thought out and patients may clash or disagree.	1001: • Champion (also chief) understands program well and can use his knowledge to gain more support from clinicians. • HBC PhD Psychologist sees a potential benefit to the P2P program in addition to the already ongoing SMAs. • Conference call to discuss concerns about P2P groups—the intention and the instructions that the P2P patients will be given; patients will be educated and should not be exchanging clinical advice—this will be covered in the do’s and don’ts’ s card and in the patient orientation. 1002: • We held a conference call with this site to go into detail about the P2P groups—the intention and the instructions that the P2P patients will be given; patients will be educated and should not be exchanging clinical advice—this will be covered in the do’s and don’ts’ s card and in the patient orientation. Team calls also clarified this. 1003: • Much more positive about the evidence behind P2P than the other sites. Absolutely see a need for the P2P program. Believe based on work with other Veteran groups that it will be very fruitful. Believe having a peer will help with attendance and motivate Veterans to attend. A previous local veteran-paired smoking group has been successful. 1005: • The ACOS is very supportive of SMAs. When introduced to PACT in 2009/2010, was introduced to concepts of SMAs – got education on SMAs and started reading about them and thought, *“Hey this is a fantastic way to actually create some efficiency in the way we provide care.”* He is a practicing PCP and has numerous diabetic patients—he has been using the clinical pharmacists and nursing staff to help manage his diabetes patients for years. • Leadership says that clinicians recognize that this will have a good impact on patients, they understand impacts on efficiency, and they understand the concepts of peer support. • Dieticians believe P2P will work because patients really enjoy having someone check up on them.
Self-efficacy Definition: Individual belief in their own capabilities to execute courses of action to achieve implementation goals.	1005: • Physician talking about engaging other providers: *“Some Primary Care providers are better than others, and I think it’s all going to have to do with their personality basically. I think some docs would be very well-suited for this where they’re not preaching at them and uh, and is okay with, like I’m kind of okay with it going off-topic every now and then but I’ll steer it back, uh, but I don’t have to be the center of attention, do you know what I mean? I don’t know, so it’s more of a Socratic method.”*	1002: • “Champion” confident he can organize the logistics for the startup of project. 1003: • P2P facilitator confident in ability to help Veterans make changes and reach goals.
PROCESS		
Planning Definition: The degree to which a scheme or method of behavior and tasks for implementing an innovation are developed in advance, and the quality of those schemes or methods.	1002: • Staff unclear of roles in SMAs – not yet defined. • Lack of schedule for SMAs.	1001: • Curriculum for SMAs tested and set prior to implementation. • Roles of clinicians in SMAs well defined prior to implementation (had nurse following up with patients for lab work, appointments, health psychologist working on goals with Vets, etc.) • Educational materials are prepared for patient use. • Nurse involved in SMAs willing to help/seek help in pairing Vets for P2P – can have group of three if pairing doesn’t work well. • Recruitment strategies thought out in terms of available patient pools. 1002: • Did SMA trial period prior to implementation. • Ready for implementation due to planning – gotten buy in at sub-site A, have organized RA. 1003: • Self-initiated local planning. The Site PI thought about ways to get buy-in from providers and planned for ways to spread the word to patients by presenting information at other groups. • P2P facilitator in place and has been attending SMAs to plan/learn more. 1004: • A lot of local planning was done. The Chief of primary care was very involved in making sure this was presented to involved staff multiple times and that buy-in from providers was obtained. 1005: • MD SMA group 2, talks about how the month interval is a good plan to follow because of the timing for when changes in behavior occur. See also knowledge and beliefs.
Opinion Leaders Definition: Individuals in an organization that have formal or informal influence on the attitudes and beliefs of their colleagues with respect to implementing the innovation.	1002: • The opinion leader for primary care was named by several staff; however, he has not been formally pulled into our project – we tried, but have not been able to yet. The named opinion leader/champion does not have the necessary influence, i.e. is not really an opinion leader. 1004: • PI and the chief of primary care seem to be opinion leaders backing this project—however, as pointed out in champions, the PI has not been able to influence 1 of the 2 SMAs groups to be supportive of this project.	1001: • The Director of primary care was named as an opinion leader; he also happens to be the champion, and a good supporter of this project. 1003: • Health Behavior psychologist is more of champion, but is also somewhat of an opinion leader for the primary care staff.
Formally appointed implementation leaders Definition: Individuals from within the organization who have been formally appointed with responsibility for implementing an innovation as coordinator, project manager, team leader, or other similar role.	1001: • Need for P2P facilitator who can engage participants. 1002: • The appointed physician implementation leader has little influence over staff at 2 of the local sites and several staff mentioned this. Also, he seems to think that he has the necessary influence, which compounds the problem. • General concern over roles in project/who will be filling roles. • P2P facilitator role of concern because seen as a lot of work/time.	1001: • RA role will be huge help for implementation of program (dedicated person to perform study related tasks). 1003: • Have P2P leaders in mind prior to implementation – is extremely engaged and prioritizes innovation.
Champions Definition: “Individuals who dedicate themselves to supporting, marketing, and ‘driving through’ an [implementation]”, overcoming indifference or resistance that the innovation may provoke in an organization.	1002: • No overall physician champion. The physicians have not bought into the diabetes SMAs. • The physicians see the diabetes SMA as extra work. They do not see the added value. • The physicians will not participate in the diabetes SMAs. Whereas, the nurses want a physician to be there for medical questions. • The named physician champion for the SMA/P2P project, does not have the influence that he needs to have (wrong champion selected) according to multiple staff.	1001: • Great champion in the director of primary care firm A. Helps with presentations, helps convince providers to enroll patients, blocks out time for providers to be at the groups, secures space, oversees local running of the project. 1002: We worked with the chief of primary care to present the study and try to gain physician buy-in for the SMAs as well as P2P groups. • Given the named champion is thought to not have adequate influence, we tried to pull in and speak with a physician who was named as being influential. 1003: • The health psychologist is a great champion for this site. He has a great deal of expertise and ideas to help with the project. He is very communicative. He is very passionate about this project and staff listen to him. 1004: • The Chief of Primary Care is a good champion for the diabetes SMAs. • Chief of PC also offered to send any emails to staff that we need him to. He said the material coming from him, would help. See also construct – leadership support. 1005: • The NP lead facilitator of most of the new SMAs is a champion, she started the SMAs (see also innovation sources), but she is also a champion. • Diabetes SMA has a nurse who was a big champion and really helped with the success of these groups according to the dietician and the lead MD facilitator. • The MD of the 2nd SMA group is a good champion and his group was well run and had good outcomes. See also innovation participants about how the PCP champion strategically used well-controlled patients in his groups.
Key stakeholders Definition: Individuals from within the organization that are directly impacted by the innovation, e.g., staff responsible for making referrals to a new program or using a new work process.	1002: • Hard sell for physicians – do not see benefit, takes too much time.	1001: • PharmDs speak at primary care meetings to educate MDs about SMA groups – overall support from clinicians for SMAs. • Champion also chief – engages key stakeholders (clinicians). 1002: • One interviewee cited a possible solution to engage residents in innovation. • One provider cited as enlisting physicians on her panel. 1004: • Chief of primary care will work to engage key stakeholders. 1005: • The ACOS for Ambulatory Care is a good supporter and has been working to engage key stakeholders in the SMAs.
Innovation participants Definition: Individuals served by the organization that participate in the innovation, e.g., patients in a prevention program in a hospital.	1001: • Poor attendance at group meetings – Vets at this site may not be comfortable in groups. • Success dependent upon engagement of participants. • Concern over finding enough interested participants. 1002: Participants need to see added value in SMA to get engagement. 1003: • Site SMAs began with recruitment of those with A1c’s over 9, attendance/engagement was very low. 1004: • Patient engagement low, patient drop-off high.	1001: • Scheduling ahead and getting reminder calls may help Vet attendance/engagement. • Pairing aspect of P2P may increase engagement among participants – peer holding them accountable. 1002: • Voluntary program – participants more likely to be motivated/engaged. 1003: • Include Vets with A1c’s under 9, which has increased attendance and engagement in program. • Having formally appointed implementation leaders (RA & P2P leader) to engage innovation participants will help. • Social support will increase engagement in SMAs. • Group setting/P2P will help to engage “passive patient population.” • Because travel is an issue, one interviewee suggested a carpool setup. 1004: • To increase engagement in SMAs/P2P, pair up Veterans at first or second SMA (previous SMA was only one visit). • Psychologists have been asking Veterans what they would like to get out of sessions/for feedback to increase engagement. • Let Veterans know they can self-refer.

Subsequent data analysis started with immersion in the data to obtain a sense of the whole and then by cataloging principal themes that emerged according to the CFIR framework constructs [17, 18]. This is a type of qualitative directed-content analysis [19] (directed initially by the CFIR constructs). Given the CFIR has 39 broad constructs related to implementation, although we analyzed, we did not discover any strong themes outside of the organizational parameters outlined in the framework.

Two authors (CK and MV) used an iterative consensus-building process to code; first each team member independently coded transcripts, and then met as a group to discuss and reconcile codes, identify emergent themes, and resolve discrepancies through consensus. Each of the CFIR constructs was ranked on a scale of − 2 to + 2 (Table 3) independently by CK and MV for each site and then were discussed until consensus ranking was reached, taking all of the qualitative data into account. The valence of each construct reflects the impact on implementation (negative or positive); the numbers provide a reference for the impact on implementation as weak or strong, with 2 being the strongest. [20]

**Table 3:** CFIR construct ranking after pre-implementation phone interviews. Rating − 2 to + 2

Construct	Site #1 (1001)	Site #2 (1002)	Site #3 (1003)	Site #4 (1004)	Site #5 (1005)
Intervention characteristics					
Intervention Source	Internal	External	Internal	External	Internal
Evidence Strength & Quality	+ 1	−2	+ 1	+ 2	+ 1
Relative advantage	−1	− 2	+ 1	+ 1	Mixed
Adaptability	−1	− 1	Missing	+ 1	Neutral
Trialability	+ 1	+ 1	Missing	Missing	Neutral
Complexity	+ 1	−2	Mixed	−1	− 2
Design Quality and Packaging	−1	− 1	− 1	−1	− 1
Cost		−1		+ 1	+ 1
Outer setting					
Patient Needs & Resources	−2	−1	−2	− 1	+ 1
Inner setting					
Networks / Communications	+ 1	−2	Neutral	Neutral	Neutral
Culture	−2	−2	Neutral	Neutral	Neutral
Tension for Change	Mixed	Neutral	+ 1	+ 1	Neutral
Compatibility	+ 1	Mixed	+ 1	Neutral	Neutral
Relative Priority	+ 1	−2	Neutral	−1	Neutral
Leadership Engagement	+ 2	−2	+ 2	+ 2	+ 1
Available Resources	−1	−1	− 1	− 1	−1
Access to knowledge & info	−1	−2	−1	− 1	−1
Characteristics of individuals					
Knowledge & Beliefs about P2P	Mixed	−1	+ 2	+ 1	− 1
Self-efficacy	Missing	Neutral	+ 1	Neutral	Neutral
Process					
Planning	+ 2	Mixed	+ 1	+ 1	Neutral
Opinion Leaders	+ 1	−1	+ 1	Mixed	Neutral
Formally appointed implementation leaders	Neutral	−1	+ 1	Neutral	Neutral
Champions	+ 2	−2	+ 2	+ 2	+ 1
Key Stakeholders	+ 1	−2	Neutral	+ 1	+ 1
Innovation Participants	−1	Neutral	−1	− 1	Neutral

## Results

Key constructs or areas of focus varied across sites. Table 2 is organized by site and CFIR construct and within that row, highlights how the findings (barrier column) informed our actions to improve or facilitate implementation (facilitator column) and what actions were taken (adaptations).

Based on our work with the participating sites to date, we identified a subset of CFIR constructs that are most likely to play a role in the implementation and possibly effectiveness of the diabetes SMAs and the P2P program, including evidence strength and quality, relative advantage, adaptability, complexity, patient needs and resources, compatibility, leadership engagement, available resources, knowledge and beliefs, and champions (Table 4). Although data from any organizational aspect mentioned by interviewees, and all CFIR constructs, were coded (see Table 2), these specific constructs formed the basis of primary qualitative analyses due to the depth and frequency of the construct throughout the interviews and qualitative analysis. A definition of each CFIR construct is included in Table 2.

**Table 4:** CFIR construct importance as ranked by local sites

All sites ranked very important in phone survey, and subsequent interview data confirmed this	All sites ranked very important or important in phone survey, and subsequent interview data confirmed this	Phone survey ranked as not very important; however, data came out strongly during the qualitative interviews
Complexity	Adaptability	Evidence Strength and Quality
Available Resources	Compatibility	Relative Advantage
Champions	Patient Needs and Resources	
Leadership Engagement		
Knowledge and Beliefs about P2P		

### Evidence strength and quality

#### Diabetes SMA

The evidence strength and quality construct constitutes stakeholders’ perceptions of the quality and validity of evidence supporting the belief that the innovation will have desired outcomes. This construct was rated positively for all sites except 1002 (see Table 2), where every interviewee mentioned that the diabetes SMAs were seen as extra work without added value or belief that the innovation will have the desired outcomes. 1002 nurse summarized these thoughts*: “I’ve got to tell you, it’s a hard sell with physicians. Even now, I don’t have a champion for the diabetes SMA. They see it as extra work. They don’t see the added value. It troubles me a lot that it’s so hard to get the docs involved.”* After this barrier was discovered, our facilitation team made a visit to present evidence and met with the local primary care team to help educate and influence the physicians.

#### Diabetes SMA and P2P

The remaining four sites’ interviews demonstrated local staff belief in the positive evidence quality for both the diabetes SMAs and P2P. A leader from site 1004 stated, “*I think [SMA and P2P] is another means for providing guidance and motivation for patients with diabetes struggling with their glucose to meet goals. I think we do a lot of telling patients what to do, and I really think there’s a benefit to hearing from ‘equals’ rather than somebody else—someone lateral as opposed to top down with the guiding and coaching.”* A Primary Care Physician (PCP) from site 1005 explained, *“No matter how much I say, ‘Yeah, I know diabetes does this, diabetes does that,’ I don’t have to deal with it and there’s one guy saying, ‘Yeah, my blood sugar’s like this, I made this little change and it dropped like, you know, dropped 20 or 30 points,’ and they believe them more.”* Sites 1001 and 1003 also mentioned that local evidence had shown that having a peer or buddy for support, as is the case in the diabetes SMAs and the P2P pairs and group sessions, helps patients with diabetes.

### Relative advantage

#### Diabetes SMA

Perceptions of the relative advantage of diabetes SMA programs were mixed across sites. Those sites that had an overall positive ranking for stakeholder’s perception of the advantage of these programs were 1003 and 1004. As an example, a 1003 health psychologist stated, *“We do have also a diabetes education class… There’s a great bit of information given. There is only a very small percentage of diabetes patients that have not taken that class because it is essentially mandated. But, we’re finding that for some reason this doesn’t translate into action.”* The need for and potential advantages of the peer support program in this population was mentioned at site 1004: *“…especially in Veteran population because they’re deployed in a unit and they come back in a unit. So the peer support would be even more effective in theory than in the general population…”.*

There were also some references from clinicians about advantages to leading group visits instead of one-on-one patient appointments and that format provided a means to hear more detailed information about patient behaviors, *“I love talking to patients but I get tired of half-hour slots, so anything that kind of breaks up my clinic, it’s a slightly different format…and it’s a chance to kind of listen to the more of the social stories and kind of what’s going in [food] why do they eat at Coney Island every day and their diet hasn’t changed, or why they’re not going to change that.”*

Sites 1001 and 1002 had negative ratings; staff did not see an advantage compared to usual care or likewise because they saw the SMAs as added worked, without any added value. For those sites with a negative ranking, we asked local staff to present information about the value and advantage to their local PCPs. During our site visits, we also presented evidence to the primary care staff from the literature, engaged them, and answered any questions they had.

#### Diabetes SMA and P2P

Another noted advantage (see Cost in Table 2) was the belief that the SMAs and P2P program will improve efficiency of care and, therefore, increase patient access and decrease cost. A physician leader from site 1004 said, *“I also think that it’s a good way to cut costs from healthcare because if a peer mentor can help the patient, he can remind him to follow his appointments, take his medications, exercise; it’s much simpler than a health professional trying to do the same thing while juggling other things. I think it helps from the clinical aspect, as well as having a financial benefit…”* A site 1005 clinical leader said, *“Let that dietician go over information with 8-12 people at one time, instead of one at a time. Those kinds of efficiencies are really great and I think it will help my dieticians, my clinical pharmacists, and my psychologist a lot, and I think it will help the physicians to manage their population of patients*.”

### Adaptabiliy

#### Diabetes SMA

Sites 1001 and 1002 had concerns initially that the SMA program was not adaptable. They thought that the overseeing site would be dictating the content and manner that each of the SMA sessions would be run. We worked with both sites continually through phone calls and virtual meetings to explain that the local team had flexibility and control over the SMA sessions and that our fidelity assessment would help to account for any differences across sites.

Site 1004 had a positive rating for adaptability of SMAs because they realized the importance of flexibility and had tailored their SMA sessions to be adaptable so Veterans could get what “*they want and need out of each session*.” Staff at this site also had a good understanding that we wanted the local clinical programs to be adaptable to fit local context.

#### P2P

Views on the potential adaptability of the peer support component of the program were mixed. Site 1001 had concerns that the program could not be adapted for patients who did not mesh well with their assigned peer. We worked with that site to come up with an adapted plan whereby a patient could be easily re-paired, even with a patient outside their cohort if need be, or assigned to a new group of 3 patients. We also implemented a way of working with the local nurses and PCPs to get their recommendations on patient pairs that would work well together. Site 1001 also had some issues surrounding their locally developed recruitment plan that the clinical and research teams thought was not feasible. During the site visit, we discussed this with the local team and they adapted their recruitment plan to be more realistic.

### Complexity

#### Diabetes SMA and P2P

Interestingly, site 1001 staff had no concerns, even when prompted, about staff being so busy that it would be difficult to do anything additional or complex. However, that was the only site with a positive ranking. The other four sites expressed concerns that if the programs were complex, this would make the programs much more difficult to implement. An Associate Chief of Staff at 1005 said, “*I am very supportive of this, but it can’t add work to my people. It will have to be very efficient*.” One specific complexity concern was in terms of the clinical notes and documentation required for each SMA visit and that the notes would be cumbersome for the clinical pharmacist, who was the documenter at one site. “*I just want the pharmacist who will be involved to write very short, patient specific changes*. *That is going to be VERY important to me. If it is burdensome to them, they are not going to be able to continue.*” The facilitation team worked with the site through team meetings and in-person discussions with the clinical pharmacists to streamline the documentation and share diabetes SMA clinical note templates across the sites.

Sites 1002, 1003, 1004, and 1005 all expressed concern with staff being busy and that it would be difficult to add additional programs and find staff time to help facilitate the SMA and/or P2P group sessions. To impact the complexity barriers, the facilitation team worked with the local sites through team meetings and phone calls to streamline documentation and shared diabetes SMA clinical note templates across the sites. Additional efforts were made to integrate the process within existing workflow--for example, allowing the clinician who documented the diabetes SMA notes to be adaptable: in some sites a clinical pharmacist, others a nurse practitioner, or primary care physician. Likewise, the role of the P2P facilitator varied.

### Patient needs and resources

#### Diabetes SMA and P2P

All sites except 1005 perceived the new programs as facing multiple barriers within the CFIR construct of addressing patient needs and resources. This was the most negatively ranked construct overall. There were a plethora of barriers across the sites, including staff concerns about patients being guarded and not wanting to pair up or exchange numbers, lack of motivation in patient population, low patient attendance especially without financial compensation for the P2P or SMA visits, difficulties with patient recruitment, long distance drives for patients to the hospital, patients’ lack of financial resources, patient resistance to change, and lack of patient follow-through when asked to bring in or complete materials or goals. A clinical pharmacist from site 1003 expressed it this way, “*There are many obstacles to bringing the patients in… [to SMAs or P2P group sessions] Our population tends to be older and on the financial scale of things, having more difficulty. Those factors set into place some natural barriers to being compliant with appointments*.”

A primary care physician at site 1001 stated, *“Definitely the top barrier will be convincing the patients to show up. We invite an average of 10 people and we usually have between 4 and 7 who come and continue to show up. I think patient buy-in is definitely a barrier.”* Site 1001 health psychologist*, “I can say generally we have a hard time getting patients to come to groups here. We’re trying to hold them first thing in the morning so that parking will be easier, but parking is a huge barrier. Patients don’t want to come to anything that they perceive as extra a lot of the time, because they find it so challenging to actually physically get here, get parked and get to their appointment.”*

Site 1004 research coordinator*, “People with diabetes don’t feel well and getting them involved in something—it is difficult. And some of our patients are still working, so if we have classes during the day, that’s an obstacle. And transportation. We’ve had that experience in the past where they’d like to join but they can’t get here.*”

To overcome these barriers the facilitation team worked to make sure that patients would be eligible for travel pay for attendance to the SMA clinical appointments and helped with attendance by making additional reminder calls and sending reminder letters. We worked with all sites to ensure that, when possible, appointments were scheduled at a convenient time for patients (also considering which time of day each facility has the most parking availability). We consulted with staff from the sites to consider their perspective on matching peers together and who would work best together and again, allowing for adaptability and patient re-pairing. We also worked with sites to ensure whenever possible that these facilitators would be sustainable across time when the research team would no longer be involved (transference of some of these tasks in time to local clinical and administrative staff).

### Compatibility

#### Diabetes SMA and P2P

Perceptions of the compatibility of the SMA and P2P programs were either mixed, neutral or positive for all sites. Site 1001 and 1003 staff were confident that the implementation process would be smooth because both programs were designed in a way that they believed was compatible and fit within their existing workflow and programs. At site 1004, we heard from multiple staff that it was very important for us to make sure the programs were integrated into their suite of already existing programs or there would be push-back from staff. To do so, we spoke with several front-line staff about how to best integrate the programs with their existing work. The Chief of Primary Care at this site also worked with us to present information at their monthly staff meetings.

Other compatibility adaptations meant that we were very flexible about the type of clinicians who could facilitate the SMAs—the main facilitators varied between clinical pharmacists, nurses, dieticians, and primary care providers; as long as multiple types of clinicians were involved, the lead was adaptable to best suit their local needs and staffing considerations. Additionally, we were flexible about the role of the P2P facilitators; Veterans with and without diabetes, research associates, and volunteers.

### Leadership engagement

#### Diabetes SMA and P2P

Leadership engagement was ranked positively at all sites except one. Leaders were considered engaged when multiple interviewees expressed that a leader did things such as: help with convincing providers to enroll patients, block out time for clinicians to facilitate the SMAs and staff to facilitate the P2P groups, guarantee space for the SMA and P2P programs to be held, garner general support from staff, help with results/outcome feedback to staff, lead from a high level, and express to staff why they feel these programs are important. At site 1002 where leadership was lacking, a named leader for the project was mentioned by local staff as not being influential. Additionally, there were some issues with general lack of high leadership support at this site (beyond the scope of this project).

### Available resources

#### Diabetes SMA and P2P

The availability of resources was ranked the same negative value across all sites (− 1). Issues of concern included space constraints for SMA and P2P group visits, lack of patient parking, SMA and P2P facilitator staff leaving after trained, staff generally busy and stretched thin, competing initiatives, overall information overload, and being short-staffed, *“We are short-staffed right now and we are unable to hire people.”* Space was so limited at site 1002 that SMA sessions had to be scheduled based on room availability, rather than staff availability.

The facilitation team helped to find guaranteed rooms, scheduled SMAs and P2P sessions early so rooms could be booked well in advance, wrote scripts for staff, and had monthly training for the P2P facilitators to ease their workload and make it easier to understand their role.

### Knowledge and beliefs

#### Diabetes SMA and P2P

There were concerns from several sites stemming from beliefs that the patients in the SMAs or as part of their pairing would share incorrect clinical information. Site 1002 health psychologist explained: “*And one other barrier potentially could be misinformation, people talk and sometimes myths get out there and misconceptions and what they hear by word of mouth which is not very accurate information so…the group sessions, we don’t know what’s going to be said between the Veterans and I think it’s important to make sure proper education being shared among them but I mean that’s going to be hard to control, so that’s another downfall*.”

Site 1005 had the most concerns about the patients being paired and chatting in the SMAs, largely based on their prior experience: “*There’s been two people that used to call each other and kind of hold each other accountable, however, they were both very non-compliant and didn’t give off the best information, so we were kind of like, ‘Eh, that didn’t work out very well.’”* This site also had a concern about peer interaction during the SMAs, as a dietician talked about a negative belief about the interaction of peers and how one could be over-bearing and change the tone. *“Then there’s this other guy that was coming to diabetes SMA and he made so many good changes, [But] he kind of came off really hard to others. He was losing weight, he was improving his A1C and we first told him, ‘Can you share your story, can you try to motivate these people?’ and it became really aggressive…someone would say, ‘No, I haven’t started exercising,’ and he’s like, ‘Why not?? Why can’t you do that?’ and it was really offending patients. We actually had to talk to him after…he was giving advice that more a provider should’ve given and it was not supportive and we had several patients call and complain about it.”*

At site 1001, 1002, 1005, we held separate conference calls, where we gave a detailed outline of the P2P program, we explained that patients would receive orientation materials and instruction and be advised not to exchange any clinical advice, and allowed staff to discuss any of their concerns with facilitators.

*“In the group [SMA] we do try to set goals each time. We’ll go over the goals that they made last month…goal-setting holds them accountable, we say, ‘Mr. so and so, did you get on the treadmill like you said you were going to last month?’ and if he hasn’t, it’s kind of like, ‘Oh, I let my group down.’ It might motivate them to try again this month and them kind of working off of each other and holding each other accountable, which is nice.”*

#### P2P

Site 1001 had what we termed a “wait and see” approach to observe if the peer program had any benefit. When prompting interviewees on their beliefs about the peer program, we were able to clear up some misperceptions and confusion about how the patients would be communicating.

Site 1003 was the most confident in the benefits of the peer program—they stated that they saw an absolute need for the peer program based on other local work with patient groups that were fruitful. They believed having a peer would help with attendance and motivate patients to attend. Furthering their confidence in the peer program, a previous local veteran-paired smoking group had been successful.

There was pushback at sites 1001, 1002, and 1005 stemming from beliefs that matching peers would be difficult for multiple reasons. Site 1001 nurse, “*Matching the patients up will probably be the hard part, I mean if one person doesn’t like their partner, I could see them wanting to stop.”*

#### Champions

Champions were ranked positively at all but one site (1002). Interestingly, the champions’ organizational role varied across sites: Director of Primary Care (3), Clinical Health Psychologist (1), and Nurse Practitioner (1).

Positive champions were defined as those working to push through any barriers and positively advocating for the clinical programs to gain staff buy-in. The Chief of Primary Care for 1004 was deemed a good champion by staff at his site. He also said, *“I see my role as making sure that primary care, as a service, sees this [SMA and P2P] as a benefit. Leading it from a high level rather and being able to tell others why we’re doing this. I can say, ‘this is important work.’”* Descriptions of what the champions did included: help with presentations, help convince providers to enroll patients, block out time for providers to facilitate/attend the groups, secure space, and oversee local management of the project.

Many interviewees at site 1002 saw their site champion as non-influential. Facilitators worked with the local site to try to gain physician buy-in for the SMAs and P2P groups. In addition, we tried to pull in and speak with a physician who was named as being influential by interviewees and staff we met on site visits.

## Discussion

In this article, we described our method for identification of contextual factors that influenced implementation of complex diabetes clinical programs -SMAs and P2P. The qualitative phone interviews aided implementation through the identification of modifiable barriers or conversely, actionable findings. Implementation projects, and certainly clinical programs, do not have unlimited resources and these interviews allowed us to determine which facets to target and act on for each site.

Likewise, our facilitation team used formative evaluation to understand the context and organizational issues at each of these VA health systems. Our approach used the CFIR to guide us to actionable findings and help us better understand barriers and facilitators, and variations of those, across constructs and sites. Using the CFIR to do this allowed us to improve the generalizability and efficiency of our findings by highlighting factors that prior research have identified as influencing implementation (each of the published constructs). Additionally, our project team and the local sites benefited from the use of formative evaluation throughout the early implementation process; we identified, in an ongoing manner, problems that we had not anticipated but that needed to be addressed to optimize implementation.

The implementation of a new clinical program is very complex and the field has recognized the need to utilize theoretical bases of implementation to facilitate implementation itself and there have been more calls for researchers to utilize existing frameworks to gain insights into the mechanisms by which implementation is more likely to succeed and to achieve common terminology. [21] We believe our approach of using the CFIR to accomplish those goals has broad applicability and can be used by other projects to guide, adapt, and improve implementation of research into practice.

We have illustrated how pre- and early-implementation FE is critically important in preparing for and gaining early understanding of key factors that influence implementation processes, and future success or failure. This early formative research shaped our implementation to minimize type III failures. Our rich examples highlight areas that were challenging as well as those that facilitated implementation of both shared medical appointments, and peer-to-peer programming. It is important to note that there was no site that was universally positive or negative across constructs, as often is assumed of “laggard” or “early adopting” sites. Evidence strength and quality was a negative issue at only one site (1002), but it was very impactful there (see Table 3, − 2 ranking) and important for the facilitators to be aware of and to work to overcome. As a result, our team presented evidence during the site visit and during a local primary care team meeting to help educate and influence the physicians. In contrast patient needs and resources was a negatively rated construct at 4 of the 5 sites, but at the 5th (1005) was a positively ranked construct—illustrating that implementation scientists need to be very cautious of labeling any construct as universally problematic.

Because of our intentional broad range of interviewees, CFIR constructs could be mixed within a site. When this was the case, the findings were discussed, weighed and used to come up with one overall score as per CFIR guidelines. The process is similar to consensus-based coding; “Analysts apply a summary rating, taking all the individual ratings and supporting qualitative summary and rationale into consideration, and then discuss ratings to achieve consensus” [22].

There are several limitations to this study. Constructs were assigned only one rating per site using weighted data from all respondents. Additionally, it is challenging for implementation researchers to identify when modifications create an additional intervention; however, in this case we classified these as local adaptations because the core underlying conceptual nature of the interventions was maintained in each case. Program drift is sometimes thought of as resulting in lower intervention success due to lack of fidelity [7]. However, experts in the field recognize that view is overly simplistic and encourages an unnecessarily rigid view of fidelity; “this designation decreased opportunities to learn from evidence-based intervention adaptations that result in improvements beyond what is expected” [10].

## Conclusions

As the SHARES study progresses, these findings will be compared and correlated to outcome measures. This comprehensive adaptation data collection will also facilitate and enhance understanding of the future success or lack of success of implementation and inform potential for translation and public health impact. Crossing the bridge from research to practice in primary care and family practice settings is crucially important because in many ways we are not reaping the full public health benefits of our investment in research. [23] While the evidence-to-practice gap for interventions in primary care is receiving attention, it tends to be understudied. [11] We believe our approach of using the CFIR to guide us to actionable findings and help us better understand barriers and facilitators, has broad applicability and can be used by other projects to guide, adapt, and improve implementation of research into practice in primary care and other clinical settings.

## Abbreviations

CFIR: Consolidated Framework for Implementation Research
FE: Formative Evaluation
IRB: Institutional Review Board
P2P: Peer-to-Peer
PCP: Primary Care Physician
SHARES: Shared Health Appointments and Reciprocal Enhanced Support
SMA: Shared Medical Appointments
VA: Veterans Affairs
